# Functional group-based linkage analysis of gene expression trait loci

**DOI:** 10.1186/1753-6561-1-s1-s117

**Published:** 2007-12-18

**Authors:** Na Li, Baolin Wu, Peng Wei, Benhuai Xie, Yang Xie, Guanghua Xiao, Wei Pan

**Affiliations:** 1 Division of Biostatistics, University of Minnesota, A460 Mayo Building MMC 303, 420 Delaware Street Southeast, Minneapolis, Minnesota 55126, USA; 2 Division of Biostatistics, Department of Clinical Sciences, University of Texas Southwestern Medical Center, 5323 Harry Hines Boulevard, Dallas, Texas 75390, USA

## Abstract

We explored approaches to using multiple related traits (gene expression levels) in linkage analysis. We first grouped mRNA transcripts according to their functions annotated in biological process of gene ontology (GO). We then compared using sample average, principal-components analysis (PCA), and linear discriminant analysis (LDA) to derive a univariate composite trait. Our results showed that PCA generally yielded stronger evidence for linkage, through the LDA component had the highest heritability. We also developed an algorithm to search for clusters of linkage peaks from multiple traits in the same group and a heuristic method for calculating *p*-value evaluating the linkage peak clustering. Future research is needed to develop rigorous methods in mapping of genes affecting the expression of a group of transcripts.

## Background

Our aim is to explore approaches to collecting information from multiple sub-clinical traits (e.g., gene expression, protein levels, metabolite measurements) in search for loci responsible for complex diseases. Given a particular regulatory/metabolic pathway that is important in the development of a disease, we use the expression levels of genes in the pathway to map the loci affecting the pathway, thus affecting the clinical outcome. To avoid confusion between the genes we are trying to map and the genes whose expression levels are used as traits, we will call the expression levels "traits" of "transcripts", as we will talk about functions of the transcripts. Because these traits reflect more immediate effects of the gene in question, we may reduce the confounding effects of environmental factors and the disease genetic heterogeneity.

Because there is no clinical trait or covariate information in Problem 1, we defined a group of transcripts as those sharing some common biological functions, and explored whether we can gain more linkage information by combining multiple expression traits in a group.

## Methods

### Selection of gene functional groups

The 3554 transcripts were grouped based on the biological processes in which they are involved according to the gene ontology (GO) via Onto-Express [1]. On the one hand, we would like to borrow information across many transcripts in a function group; on the other hand there is an increasing functional heterogeneity with a larger or less-specific function group. To achieve a trade-off between a functional specificity and group size, and to ensure that the results from several groups are comparable, we restricted our analysis to those groups with approximately 10 to 20 transcripts. We calculated pairwise correlations between pairs of traits in the same group and the general heritability was estimated using SOLAR [2] for each of the 554 transcripts in 40 groups. The top 10 groups with the highest average heritability estimates were selected for subsequent linkage analysis because they were more likely to contain interesting linkage signals.

### Linkage analysis with composite trait

All traits were standardized to have sample mean of 0 and sample variance of 1. We used three approaches to producing a univariate summary of the expression traits of individual genes in each group: a sample average, principal-components analysis (PCA), and linear discriminant analysis (LDA). All three approaches derive a single or multiple univariate "composite" traits by using a linear combination of the individual traits. The components in PCA are orthogonal linear combinations of original data and are ordered by decreasing sample variances [3]. In particular, the first component explains the largest proportion of sample variation. PCA was carried out using function *prcomp *in R. The PCA did not take into account the fact that the subjects came from several distinct families. As an alternative, we sought to find a linear combination of the original data that maximized the ratio of inter-family variance to within-family variance, for which we used LDA with the family as the class label. The LDA was carried out using function *lda *in R.

Multipoint variance-component LOD scores for each transcript and composite traits were calculated using Merlin [4].

### Combining linkage results from multiple traits

In linkage studies in which multiple related traits (such as obesity, diabetes, and hypertension) are analyzed, it is often of interest to see if several of the traits have linkage signals around a common region, often done by simply visualizing the LOD scores along a chromosome. We developed a heuristic algorithm for identifying the clustering of linkage peaks. 1) Linkage "peaks" were defined as LOD scores greater than a particular threshold *C *(e.g., 2). The threshold was set to be relatively high such that the chance of type I error was low. 2) The peak locations were defined to be where the local maximum LOD scores were. 3) Using a sliding window with width *W *(e.g., 10 cM), we defined a "cluster" as the window inside which more than one distinct gene had one or more peaks.

To assess whether a cluster was due to chance, we calculated a simple *p*-value, assuming that linkage peaks were independent and uniformly distributed along a chromosome, conditional on the observed number of peaks for each gene. Let *L *denote the total chromosome length. For gene *k*, conditional on observing *n*_*k *_peaks, the probability of observing at least one peak in a window is $p_{k}=1-{\left(1-W/L\right)}^{n_{k}}$. We calculated the average probability as

$$\bar{p}=\int_{k=1}^{K}p_{k}/K=1-\frac{1}{K}\int_{k=1}^{K}{\left(1-W/L\right)}^{n_{k}}.$$

For the total *K *transcripts in a group, the probability that there are at least *K*_0 _peaks in a window is

$$\int_{m=K_{0}}^{K}\left(\begin{array}{c} K\\ m \end{array}\right){\bar{p}}^{m}{\left(1-\bar{p}\right)}^{K-m}.$$

## Results

### Selection of functional groups

**Figure 1:**
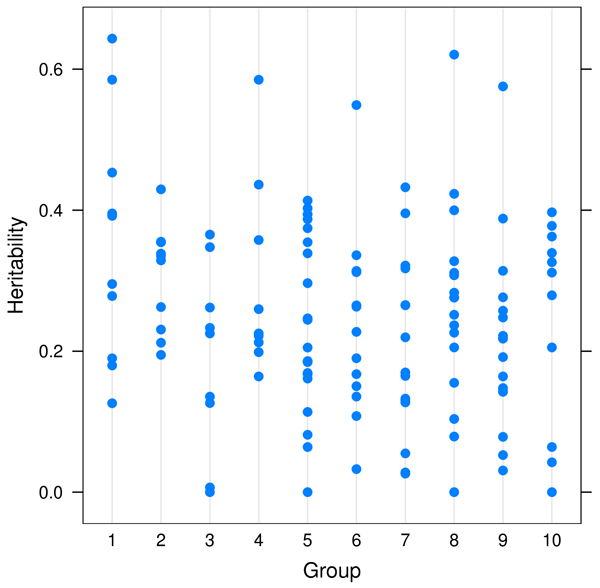
**Heritability of the ten functional transcript groups with highest mean heritability**. The groups are: 1) amino acid biosynthesis; 2) phosphoinositide-mediated signaling; 3) GTP biosynthesis; 4) purine nucleotide biosynthesis; 5) regulation of cyclin dependent protein kinase activity; 6) meiosis; 7) mRNA-nucleus export; 8) cholesterol metabolism; 9) biosynthesis; and 10) epidermis development.

**Figure 2:**
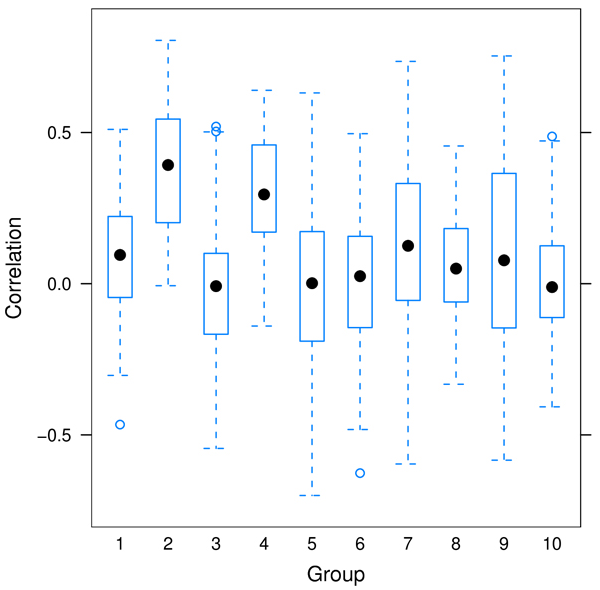
Pairwise correlations of the ten functional groups with highest mean heritability.

Figure 1 shows the heritability of the ten selected groups. The median heritability varies from 0.17 to 0.39. The pairwise correlation of traits within each group is shown in Figure 2. Some groups show strong positive correlations (e.g., Groups 2 and 4), while for others the correlation was distributed almost symmetrically around 0 (e.g., Groups 5 and 6). Our definition of function groups does not necessarily imply the transcripts are co-regulated directly or indirectly through the same gene or pathway so it is not surprising to see that some transcripts within a group have very little correlation.

### Linkage analysis with composite traits

**Figure 3:**
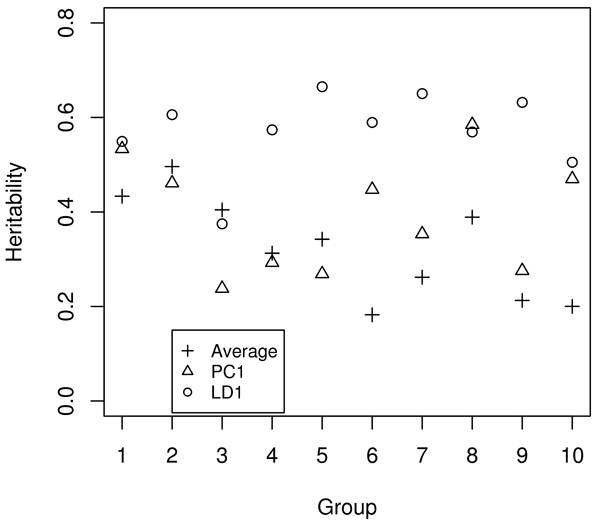
Heritability of the derived composite traits.

We calculated the LOD scores for individual transcripts and composite traits for the ten functional groups. Generally the first principal component (PC1) captured the common LOD peaks in individual traits well, and sometimes the PC1 had higher LOD scores than any of the individual traits but it might miss a high LOD score that would be obtained from just one trait. The sample average performed similar to or slightly worse than PC1, while LD1 (first discriminant in LDA) was the least capable of recovering and enhancing the single-trait LOD score peaks, despite the fact that LD1 always had the highest heritability, as expected, as a result of its construction (Fig. 3).

**Figure 4:**
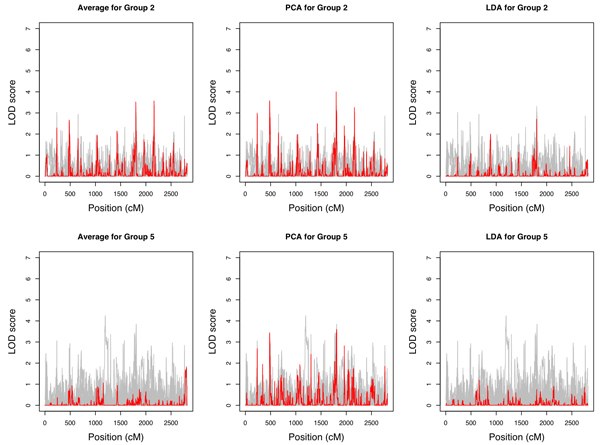
**LOD scores**. Individuals genes (gray) and the composite traits (red) for groups 2 (phosphoinositide-mediated signaling) and 5 (regulation of cyclin dependent protein kinase activity).

The LOD score curves for individual transcripts and composite traits for Groups 2 and 5 are shown in Figure 4. In both cases, PC1 performed well in terms of yielding the strongest evidence for linkage compared with single-trait analysis, while the sample average did poorly for Group 5; it was likely due to the fact that transcripts in Group 2 were mostly positively correlated, whereas about a half of the transcripts in Group 5 were negatively correlated with the other half. PC1 accounted for 45% and 26% of the total variances for Groups 2 and 5, respectively.

### Combining linkage results from multiple traits

Table 1 summarizes the clustering of linkage peaks for Groups 2 and 5. Without any multiple testing adjustment, it appeared that in general the probability of chance clustering was low even when only two peaks appeared in the same window. If a strong clustering were observed (e.g., three or more peaks clustered together out of ten genes), it could be a strong indicator of the existence of a possible common regulatory locus (co-linkage).

**Table 1:** Clustering of LOD score peaks

				Clusters^a^	
				
Group	No. genes	LOD threshold	Total no. of peaks	2-peak (*p*-value)	3-peak (*p*-value)
2	11	2	18	3 (0.002)	1 (3 × 10^-5^)
		3	4	1 (10^-4^)	0
5	21	2	49	5 (0.01)	2 (7 × 10^-4^)
		3	14	1 (10^-3^)	0

^a^The last two columns show the number of clusters with two (or three) peaks with the corresponding *p*-values (i.e., probability of having two (three) or more peaks in a window) in parentheses. A LOD score threshold value of 2 or 3 was used to define a peak.

## Conclusion

Li et al. [5] used the average expression profile of a transcription module as a quantitative trait in linkage mapping and found that it was more powerful than using individual expression traits. Lan et al. [6] also used PCA and hierarchical clustering seeded by relevant disease traits for dimension reduction in expression quantitative trait locus mapping, but they did not emphasize its use for a functional group. Here, we compared the performance of several dimension reduction techniques and found PCA generally outperformed the sample average when applied to GO-defined functional groups, at least in terms of capturing and enhancing any linkage scores as obtained from a single-trait analysis. The motivation behind using LDA was to maximize the heritability of the derived trait because the heritability is approximately the ratio of intra-versus inter-family variances. However, the linkage result for the LDA-derived trait was actually the worst. The LOD scores are generally smaller than those from single-trait analysis and for Group 5, almost all of the LOD score peaks from single-trait analysis were gone, suggesting that it is not always desirable to search for traits with high heritability.

We selected the ten groups for linkage analysis based on the heritability. There is substantial difference in the correlation structure within each group as evidenced in Figure 2. It is not clear for what correlation structure such combined analysis is likely to be the most successful. On the one hand, traits that are correlated are more likely to share a common regulatory gene. On the other hand, two highly correlated traits do not provide much additional information compared with a single trait [7].

Although using PCA does not necessary yield higher LOD scores than a single expression trait, different thresholds for "significant" LOD scores are necessary due to multiple testing when multiple traits are analyzed individually. A dimension reduction approach necessarily reduces the number of tests conducted. We could, for example, use PCA to screen for functional groups that are more likely to be co-regulated by a common gene among all GO groups.

The way we searched for clustering of linkage peaks among related traits was just a proof-of-concept exercise. In particular, information such as the width or height of a linkage peak was not used. Our *p*-value calculation was based on perhaps over-simplified assumptions, such as the independence of peak locations under the null hypothesis. Much further research is needed.

Finally, given an overwhelmingly large set of variables (e.g., gene expression levels), how to define functional groups will likely be the most critical part of the analysis. We realized that the GO functional groups do not necessarily imply the transcripts in a group belong to the same metabolic pathway or are regulated by common genes. Without sufficient biological knowledge and no clinical outcomes, we could not address the problem of how to select traits for joint analysis and opted to simply use GO information. Solutions to this difficult problem are highly context dependent and close collaboration between statisticians and subject-area experts is needed.

## Competing interests

The author(s) declare that they have no competing interests.
